# Poster Session I - A176 MANTLE CELL LYMPHOMA DIAGNOSED ON FIT-POSITIVE COLONOSCOPY: A CASE REPORT AND REVIEW OF GASTROINTESTINAL LYMPHOMA

**DOI:** 10.1093/jcag/gwaf042.176

**Published:** 2026-02-13

**Authors:** M Saunders, N Fu

**Affiliations:** The University of British Columbia, Vancouver, BC, Canada; The University of British Columbia, Vancouver, BC, Canada

## Abstract

**Background:**

Lymphoma is an uncommon etiology of colorectal malignancy within the gastrointestinal (GI) tract comprising 0.2% of all colorectal malignancy. Among GI lymphomas, only 10-20% involve the colon, whereas the majority involve the stomach (65%) or small intestine (20-30%).

**Aims:**

A 56-year-old asymptomatic male with obesity and obstructive sleep apnea on no medications without prior endoscopies underwent a colonoscopy for a positive Fecal Immunohistochemical Test (FIT).

**Methods:**

He had no family history of GI malignancies or lymphoma. Other baseline laboratory work was unremarkable, including a complete blood count, metabolic panel, and iron indices. Colonoscopy revealed a vascular lesion in the cecal base measuring over 5 cm and a 2cm pedunculated ascending colon polyp removed via hot-snare polypectomy. Biopsies of the cecal mass and removed polyp displayed unremarkable colonic mucosa with prominent underlying expanded submucosal lymph nodes. Histological assessment of the predominant small B-cells revealed positivity to CD5, CD20, BCL2, and Cyclin-D1 diagnostic for Mucosa-associated Lymphoid Tissue Lymphoma, Mantle Cell Lymphoma (MCL). Staging CT scan revealed multifocal lymphadenopathy, splenomegaly, and a dominant intraluminal cecal mass. The patient was subsequently induced on bendamustine and rituximab chemoimmunotherapy with three cycles tolerated well to date.

**Results:**

Colorectal lymphomas include MCL, Diffuse Large B-cell Lymphoma, and Burkitt’s Lymphoma. Presenting symptoms are non-specific such as diarrhea, rectal bleeding or abdominal pain. MCL, which is a subtype of B-cell non-Hodgkin Lymphoma defined by over expression of cyclin D1 and CD5/20, accounts for less than 4% of primary GI lymphomas. Endoluminal characteristics of MCL are variable and may include ulcerated mucosa, solitary lesions, mucosal protrusions, and multiple lymphomatous polyposis.

The primary diagnosis of MCL in this case was made histologically via colonic biopsy and polypectomy obtained during a FIT-positive colonoscopy. FIT testing was positive likely due to the presence of the large, vascular cecal MCL lesion. Early identification through short colonoscopy wait times for FIT positivity allowed for an expedited diagnosis and timely initiation of appropriate systemic treatment for MCL, which is crucial for disease prognosis.

**Conclusions:**

Mantle Cell Lymphoma is a rare B-cell lymphoma that can present as an incidental finding on routine colonoscopy. This case emphasizes the often subtle and variable presentation of GI lymphoma. It also highlights the importance of including hematologic malignancies such as MCL in the differential diagnosis of atypical or submucosal colonic lesions. Multidisciplinary collaboration was essential in this case for expediting diagnosis confirmation, treatment planning, and long-term follow-up.

**Funding Agencies:**

None

